# Nanosilk Increases the Strength of Diabetic Skin and Delivers CNP-miR146a to Improve Wound Healing

**DOI:** 10.3389/fimmu.2020.590285

**Published:** 2020-10-30

**Authors:** Stephen M. Niemiec, Amanda E. Louiselle, Sarah A. Hilton, Lindel C. Dewberry, Liping Zhang, Mark Azeltine, Junwang Xu, Sushant Singh, Tamil S. Sakthivel, Sudipta Seal, Kenneth W. Liechty, Carlos Zgheib

**Affiliations:** ^1^Laboratory for Fetal and Regenerative Biology, Department of Surgery, University of Colorado Denver School of Medicine and Children’s Hospital Colorado, Aurora, CO, United States; ^2^Department of Materials Science and Engineering, Advanced Materials Processing and Analysis Center, Nanoscience Technology Center, University of Central Florida, Orlando, FL, United States; ^3^College of Medicine, UCF Prosthetics Cluster, University of Central Florida, Orlando, FL, United States

**Keywords:** nanosilk, diabetic wound, microRNA-146a (miRNA-146a), cerium oxide nanoparticle (CNP), wound healing

## Abstract

Diabetes mellitus is a metabolic disorder associated with properties and an increased risk of chronic wounds due to sustained pro-inflammatory response. We have previously of radical scavenging cerium oxide nanoparticles (CNP) conjugated to the anti-inflammatory microRNA (miR)-146a, termed CNP-miR146a, improves diabetic wound healing by synergistically lowering oxidative stress and inflammation, and we sought to evaluate this treatment in a topical application. Silk fibroin is a biocompatible polymer that can be fabricated into nanostructures, termed nanosilk. Nanosilk is characterized by a high strength-to-density ratio and an ability to exhibit strain hardening. We therefore hypothesized that nanosilk would strengthen the biomechanical properties of diabetic skin and that nanosilk solution could effectively deliver CNP-miR146a to improve diabetic wound healing. The ability of nanosilk to deliver CNP-miR146a to murine diabetic wounds and improve healing was assessed by the rate of wound closure and inflammatory gene expression, as well as histologic analysis. The effect of nanosilk on the properties of human diabetic skin was evaluated by testing the biomechanical properties following topical application of a 7% nanosilk solution. Diabetic murine wounds treated with topical nanosilk and CNP-miR146a healed by day 14.5 compared to day 16.8 in controls (p = 0.0321). Wounds treated with CNP-miR146a had higher collagen levels than controls (p = 0.0126) with higher pro-fibrotic gene expression of TGFβ-1 (p = 0.0092), Col3α1 (p = 0.0369), and Col1α2 (p = 0.0454). Treatment with CNP-miR146a lowered pro-inflammatory gene expression of IL-6 (p = 0.0488) and IL-8 (p = 0.0009). Treatment of human diabetic skin with 7% nanosilk solution resulted in significant improvement in maximum load and modulus (p < 0.05). Nanosilk solution is able to strengthen the biomechanical properties of diabetic skin and can successfully deliver CNP-miR146a to improve diabetic wound healing through inhibition of pro-inflammatory gene signaling and promotion of pro-fibrotic processes.

## Introduction

Diabetes mellitus (DM) is a metabolic disorder affecting over 34.2 million people in the United States, with the incidence continuing to rise (1). The hyperglycemia associated with DM raises the risk of cardiovascular disease, renal failure, and neuropathy (2–4). Diabetic patients are particularly susceptible to skin injury and chronic wound formation with a 25% lifetime risk of developing a foot ulcer; within the United States, diabetic foot ulcers are the indication for 84% of all amputations and carry a cost of over $9 billion (5–7). There is a clear need for therapeutic developments in the care of chronic diabetic wounds. The purpose of this study is to evaluate the role of nanosilk solution as a topical delivery system for a novel therapeutic, Cerium Oxide Nanoparticle-microRNA146a (CNP-miR146a), in the treatment of diabetic wounds.

Normal wound healing is characterized by early recruitment of inflammatory cells, particularly neutrophils. Neutrophils release important cytokines, such as transforming growth factor β1 (TGF-β1), monocyte chemoattractant protein 1, and fragments of extracellular matrix (ECM) proteins in order to recruit monocytes (8). Once monocytes penetrate the wound site, they are activated into pro-resolving macrophages that secrete growth factors such as transforming growth factor-β1 (TGF-β1), fibroblast growth factor (FGF), platelet derived growth factor (PDGF), and vascular endothelial growth factor (VEGF) which are essential to initiating tissue formation (8, 9). In the final stages of healthy wound healing, fibroblasts are activated by TGF-β1 into myofibroblasts, which contribute to wound contraction (10, 11).

Diabetic wounds are characterized by a reduced production of growth factors, abnormal angiogenesis, and decreased collagen deposition (12–14). While the impairment in diabetic wound healing is multifactorial, a critical component in its pathophysiology is chronic inflammation and maintenance of a pro-inflammatory macrophage state, ultimately leading to poor wound contraction and failure of scar formation (14–16). Diabetic wounds are characterized by increased expression levels of pro-inflammatory cytokines interleukin (IL)-6 and IL-8 (15, 16). We have previously shown that intradermal injection of CNP-miR146a in murine and porcine models is able to accelerate the wound healing process through reduction of the inflammatory response (17).

CNP-miR146a is the conjugation of cerium oxide nanoparticles (CNP) to microRNA-146a (miR146a). Cerium oxide is a naturally occurring metal characterized by dual valency with 3+ and 4+ states and is known to have free radical scavenging properties (18–23). The *in vivo* and *in vitro* application of these CNPs have been found to be highly effective in ameliorating chronic inflammation and oxidative stress (18, 24–27). miR146a is an anti-inflammatory microRNA known to inhibit the Nuclear Factor kappa-light-chain-enhancer of activated B cells (NF-κB) pro-inflammatory signaling pathway, a key activator of IL-6 and IL-8 (28, 29). We have previously shown the conjugate treatment of intradermal CNP-miR146a to be superior to the individual components, CNP and miR146a, in accelerating diabetic wound closure (17), and we sought to evaluate a topical application of this conjugate therapy through delivery in a nanosilk solution.

Silk fibroin is a biodegradable and biocompatible polymer, which can be fabricated into different nanostructures for biomedical application. Nanosilk is a highly tensile biomaterial with a strength-to-density ratio higher than steel. It is able to exhibit strain hardening and is thought to be tougher than Kevlar (30). We have previously shown that diabetic skin biomechanically inferior to normal skin, therefore at increased risk for breakdown and wounding with delayed healing (31). Therefore, if silk fibroin solution can act as a topical delivery system for novel therapeutics, it could potentially have the added benefit of biomechanical protection during the healing process. We hypothesized that topical application of nanosilk solution could effectively deliver CNP-miR146a to improve murine diabetic wound healing and that nanosilk solution would further strengthen the biomechanical properties of uninjured diabetic skin.

## Materials and Methods

### Manufacturing of Nanosilk

Nanosilk fibroin solution was prepared per previously reported protocols (32). Briefly, silk cocoons isolated from *Bombyx mori* silk worms (Technical grade, Purchased from Aurora Silk, USA) were weighted 5 g and degummed using boiling water for 30 min containing 0.02M Na_2_Co_3_. The degummed silk was washed three times and allowed to dry out completely. Once dry, the silk was incubated 60°C for 4 h in lithium bromide solution which produces an amber-colored, highly viscous nanosilk fibroin solution. This solution is further dialyzed using ultrapure water for 48 h and filtered of impurities by centrifuging at 9,000 rpm. Finally, a clear and amber colored aqueous nanosilk solution was obtained with average nanosilk fibroin concentration ranging from 6 to 8% (weight/volume) and stored at 4°C for further experimental use. The nanosilk was characterized for its size and zeta surface charge using Zeta Sizer Nano (Malvern Instruments).

### Synthesis and Characterization of CNP-miR146a

Cerium oxide nanoparticles were synthesized using simple wet chemistry, as described previously (32, 33). Briefly, cerium nitrate hexahydrate (99.999%, Sigma Aldrich) was mixed with deionized water. Hydrogen peroxide was added to the mixture forming crystalline nanoparticles of cerium oxide immediately upon oxidation. This CNP was used for conjugate synthesis with miRNA146a molecule using the CDI (i.e. 1,1-carbonyldiimidazole) chemistry, as described previously (17). The CNP-miRNA146a conjugate was dialyzed to remove the unbound molecules using DNA/RNAse free water and stored at −80°C until use. Hydrodynamic size and zeta surface charge were analyzed using Zeta Sizer Nano (Malvern Instruments). The morphologies of CNP and CNP-miR146a were further characterized by FEI Tecnai F30 Transmission Electron Microscopy (TEM) at accelerating voltage of 300 kilovolts (kV).

### Animal Model of Diabetic Wound Healing

Care of study animals were in compliance with guidelines outlined in the NIH Guide for the Care and Use of Laboratory Animals. All experimental protocols were reviewed and approved by the Institutional Animal Care and Use Committee at the University of Colorado Denver–Anschutz Medical Campus (License #84-R-0059). Fourteen- to 18-week-old female diabetic mice (BKS.Cg-Dock7m+/+Leprdb/J, strain No. 000642, Jackson Laboratory) were used for this study. All mice were confirmed to have a blood glucose level greater than 300 mg/dl. All wounding procedures were performed under inhaled anesthesia with Isofluorane and mice received a single subcutaneous injection of Buprenorphine for post-procedural analgesia (Schering-Plough Animal HealthCorp). The posterior neck and back were shaved and depilated prior to wounding. The area was cleaned with an alcohol swab and a single dorsal full-thickness wound was made with an 8 mm punch biopsy (Miltex, Inc.). Wounds were treated with topical application of 50 μl of phosphate buffered saline (control), nanosilk alone (NS), or nanosilk with CNP-miR146a (NS + CNP-miR146a, n = 4–5 per group). Wounds were then dressed with a Tegaderm (3M), which was subsequently removed on post-operative day 2.

### Duration and Rate of Wound Healing

Wounds were photographed every other day until complete closure. ImageJ (National Institutes of Health; http://rsbweb.nih.gov.proxy.hsl.ucdenver.edu/ij/) was used to measure the wound area. After complete closure, wounds were harvested for downstream analysis as described below.

### Immunohistochemical Evaluation of Fully Healed Wounds

After full wound closure, the area of scar was excised and half the wound was fixed in 10% formalin, with the remaining wound used for biochemical analysis. After 24 h of formalin fixation, wounds were placed in 70% ethanol solution prior to paraffin embedding. Paraffin embedded tissue was sectioned at 5 μm, then stained with Masson’s trichrome. Ten random high-powered fields (HPF, 400× magnification) were imaged of trichrome-stained slides along the healed wound edge. Using an automated algorithm on NIS Elements–Advanced Research imaging software, the area of blue, representing collagen, per HPF was quantified and averaged for each sample.

Slides used for immunohistochemistry were deparaffinized and placed in a citrate buffer (pH 6.0). The heat-induced epitope was retrieved with the Decloaker (Biocare Medical) and stained (Leica’s Bond Rx). CD45 is a common leukocyte antigen present on all white blood cells. Primary CD45 antibodies at 1:50 solutions (BD Biosciences, Cat #610265, RRID: AB_397660) were applied to slides, which were then developed with a Vectastain Elite ABC kit (Vector Laboratories). CD45-stained slides were analyzed and quantified with NIS Elements–Advanced Research imaging software for the relative area of CD45-positive cells, representing leukocytes, per HPF (400× magnification) by evaluating ten consecutive high-powered fields along the wound edge. A second set of wounds harvested at 7 days after injury were evaluated for CD45 count as described above.

### Polymerase Chain Reaction to Measure Relative Gene Expression in Wounds After Treatment

Fully healed wounds were immediately placed into liquid nitrogen and then frozen at −80°C until processing for total RNA extraction. Skin samples were homogenized in Qiazol (Qiagen) and processed for RNA extraction per manufacturer’s instructions. Isolated RNA was treated with DNAse I (Thermo Fischer Scientific), converted to cDNA (Applied Biosystems RT Kit), and used for reverse transcriptase amplification using the BioRad CFX-9600 thermal cycler. Normalization was attained using the housekeeper gene β-actin. Expression analysis of Col1α2 and Col3α1 was performed in triplicate and the average of each triplicate was used for statistical analysis of each sample. To evaluate the early gene expression profile in healing wounds, a second subset of mice were wounded as above and sacrificed 7 days after injury. Wound edge was harvested and processed for RT-qPCR as described above. PCR analysis of IL-6, IL-8, and TGFβ-1 was performed in triplicate and the average of each triplicate was used for statistical analysis. Again, β-actin was used for normalization.

### Biomechanical Testing of Nanosilk on Human Diabetic Skin

Murine diabetic skin is biomechanically inferior to non-diabetic skin at baseline (31). To evaluate human diabetic skin, human skin samples were obtained from the National Disease Research Interchange (NDRI) protocol “DLIK3 001 006 – Type 2 Diabetes Full Thickness Skin.” The protocol includes patients 45 to 70 years old. Skin samples were collected from intact skin over the anterior tibia, preserving cranial-caudal orientation. Samples were treated topically with 10 ul of either control solution (PBS) or 7% nanosilk solution. The solution was allowed to dry completely—approximately 5 min—prior to biomechanical testing with Instron 5942 testing unit. A laser-based device was first used to measure cross-sectional area for use in calculations of modulus and maximum stress. Samples were fixed to the testing unit using a cyanoacrylate adhesive to prevent slipping. Each testing unit was placed in a heated PBS bath prior to preloading to 0.0005 Newtons (N). The sample was held at this preload for 120 s, then subjected to gradual increases in force until the sample tore, indicating sample failure in holding tension. Local tissue strain was measured optically. Maximum stress at failure was calculated using linear regression of the stress-strain curve. Modulus of elasticity was determined by the slope of the stress-strain curve. Data and analyses for biomechanical testing was performed with Bluehill 3 software.

### Statistical Analysis

Analysis of quantitative data was performed using one-way ANOVA with a two-sided significance level of α = 0.05 with GraphPad Prism 8 software (La Jolla, CA, USA). For differences in wound closure across time, a two-way ANOVA was performed to compare mean wound closure between groups at each measured timepoint. For all analyses, the mean of each treatment group was compared to the mean of the control group.

## Results

### Characterization of Nanosilk and CNP-miR146a

**Figure 1:**
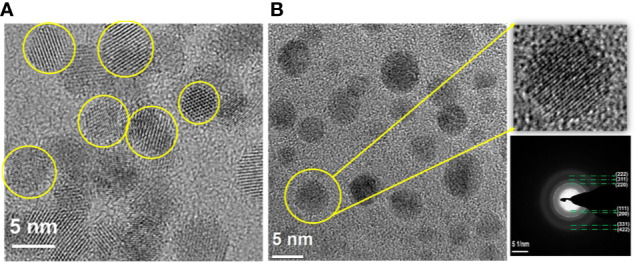
Morphological characterization of nanostructures, **(A)** CNP and **(B)** CNP-miR146a. **(A)** Well crystalline spherical shape control CNP particles in the size of 3–5 nm. **(B)** CNP-miR146a conjugates with a stabilized and dispersed spherical particle of 3–7 nm size, with a very thin coating (dark shading) on the nanoparticles, showing the conjugated miR146a. The selected area electron diffraction (SAED) pattern [(111), (200), (220), (311), (222), (331), and (422)] confirms the diffraction rings match the cubic structure of ceria.

The nanosilk solution was found to be at 7% concentration with an average diameter of 11.97 nm and zeta surface charge of −3 millivolts (mV). The CNP and CNP-miR146a conjugate were also found to be in the respective nanometer zone, at 20 nm and 190 nm, respectively. The hydrodynamic radius and surface zeta charge of CNP (20 nm, 27 mV) was found to be altered significantly due to the surface conjugation of miR146a molecules (CNP-miR146a: 190 nm, −18mV). **Figure 1** demonstrates the morphological characterization of CNP before and after miR146a conjugation. The particles are spherical in shape with a 3 to 7 nm size both before and after conjugation.

### Nanosilk Delivery of CNP-miR146a Improves Diabetic Wound Healing

**Figure 2:**
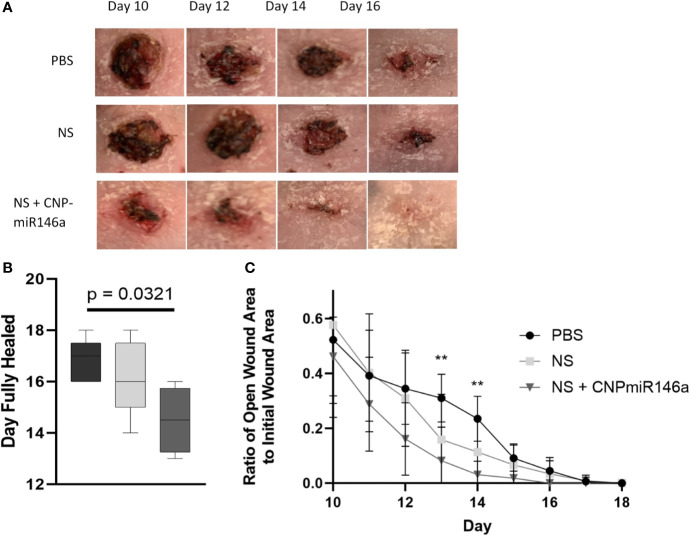
**(A)** Representative images of diabetic murine wounds during the healing process after PBS, NS, or NS + CNP-miR146a topical treatments. **(B)** Diabetic wounds closed significantly faster when treated with NS + CNP-miR146a. **(C)** Wounds were significantly smaller 13 and 14 days after wounding following treatment with CNP-miR146a compared to PBS-treated controls 14 days after treatment (**p < 0.05).

Murine dorsal wounds, as described above, were allowed to heal completely with pictures taken daily to measure wound size until full closure (**Figure 2A**). Diabetic wounds treated with PBS healed at day 16.8, while wounds treated with NS + CNP-miR146a healed significantly faster at days 14.5 (p = 0.0321, **Figure 2B**). During the wound healing period, wounds treated with PBS had closed to 30.99% and 23.48% of the original size at days 13 and 14, respectively. At days 13 and 14, wounds treated with NS + CNP-miR146a were significantly smaller at 8.216% (p = 0.0419) and 3.097% (p = 0.0051) of the original size (**Figure 2C**).

### Nanosilk With CNP-miR146a Increases Collagen in Healed Diabetic Wounds

**Figure 3:**
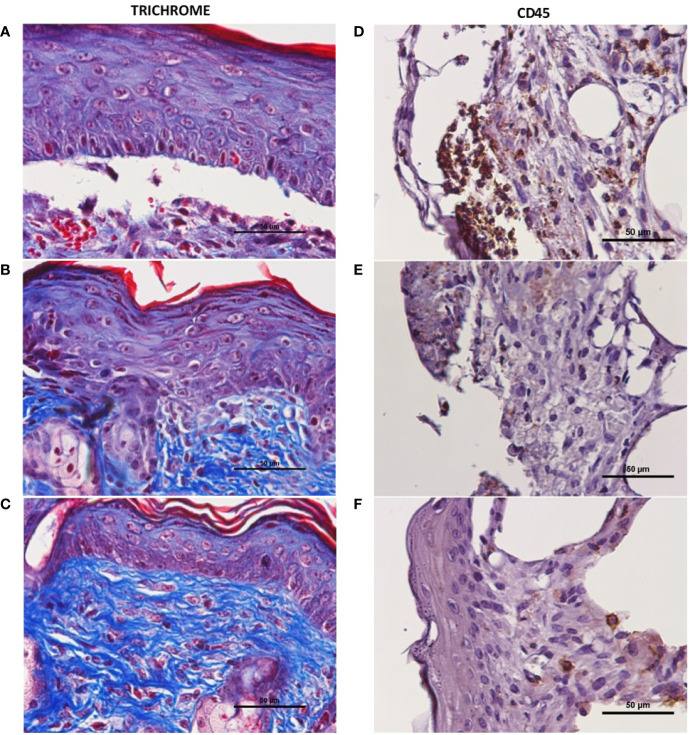
Representative histology of fully healed diabetic wounds. There was an increase in collagen level (blue stained tissue in trichrome) after treatment with NS + CNP-miR146a **(C)** compared to those treated with PBS alone **(A)**. **(A–C)** 400× magnification of trichrome-stained fully healed wounds. **(D–F)** 400× magnification of CD45+ stained wounds 7 days after wounding. **(A, D)** Diabetic wounds treated with PBS. **(B, E)** Diabetic wounds treated with nanosilk solution. **(C, F)** Diabetic wounds treated with nanosilk solution carrying CNP-miR146a.

**Figure 4:**
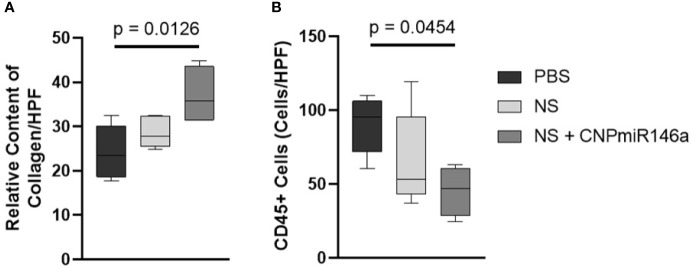
**(A)** Average collagen level per 400× magnification high-powered field (HPF) was higher in fully healed wounds when wounds were treated with NS + CNP-miR146a. **(B)** Average CD45+ cell count per 400× magnification HPF was lower in wounds treated with NS + CNP-miR146a 7 days after injury.

Wounds were allowed to fully heal and processed for histologic analysis as described above. Trichrome-stained slides are depicted in **Figures 3A–C**, with blue stain selecting for collagen. When quantified per HPF, there was a significant increase in collagen level per HPF at the time of full heal when wounds were treated with NS + CNP-miR146a compared to control diabetic wounds (p = 0.0126, **Figure 4A**). CD45 stains for leukocytes, as depicted in **Figures 3D–F** of wounds at 7 days after injury. There was no significant difference in CD45 cell counts per HPF between treatment groups at the time of full heal (not pictures), however, CD45 count at 7 days after wounding was significantly lower in wounds treated with NS + CNP-miR146a compared to control wounds (p = 0.0454, **Figure 4B**).

### CNP-miR146a Effectively Increases Pro-Fibrotic Gene Expression in Diabetic Wounds

**Figure 5:**
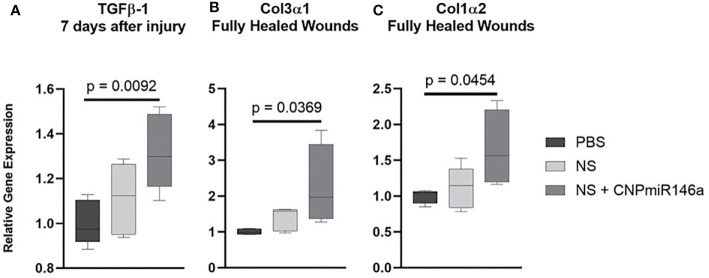
Relative gene expression of pro-fibrotic genes after wounding in diabetic mice. **(A)** Mice treated with CNP-miR146a had higher expression of TGFβ-1 7 days after injury when compared to PBS-treated wounds. **(B–C)** Fully healed wounds had higher relative expression levels of Col3α1 and Col1α2 when treated with CNP-miR146a.

Tissue was collected both at 7 days following injury and at time of full heal to evaluate relative gene expression of pro-fibrotic genes. Early in the healing process at 7 days, there was a significant increase in TGFβ-1 gene expression following treatment with NS + CNP-miR146a compared to controls (p = 0.0143, **Figure 5A**). At 7 days, there was no significant difference between treatment groups in expression of Col3α1 or Col1α2. However, once fully healed, mice treated with CNP-miR146a in nanosilk solution had significantly higher Col3α1 and Col1α2 gene expression compared to PBS-treated mice (p = 0.0369 and p = 0.0454, respectively, **Figures 5B–C**).

### CNP-miR146a Modulates Pro-Inflammatory Gene Signaling During Diabetic Wound Healing

**Figure 6:**
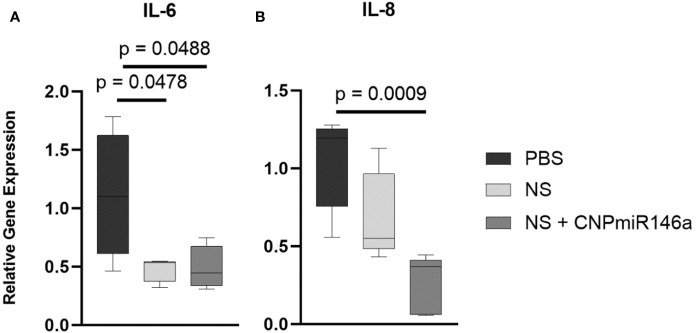
CNP-miR146a, when delivered topically with nanosilk solution, significantly lowers early pro-inflammatory gene expression of IL-6 **(A)**, p = 0.0488) and IL-8 **(B)**, p = 0.0009) 7 days after injury. Nanosilk alone also lowered IL-6 gene expression **(A)**, p = 0.0478) 7 days into healing.

To evaluate the mechanism by which CNP-miR146a may speed diabetic wound healing and promote collagen deposition, we evaluated the gene expression of pro-inflammatory IL-6 and IL-8 during the healing process by harvesting the wound edge 7 days after injury. As depicted in **Figure 6**, wounds treated with nanosilk alone lowered IL-6 relative gene expression compared to PBS-treated wounds (p = 0.0478). The conjugate treatment NS + CNP-miR146a significantly lowered both IL-6 and IL-8 gene expression seven days after wounding (p = 0.0488 and p = 0.0009, respectively).

### Nanosilk Solution Improves the Biomechanical Properties of Human Skin

**Figure 7:**
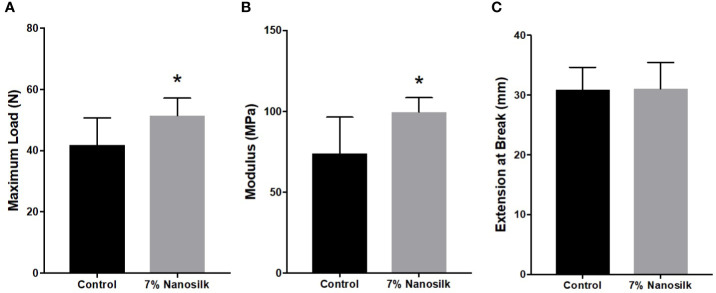
Biomechanical properties of human diabetic skin. Human skin samples were treated with 10 ul of either PBS (control, n = 8) solution or 7% Nanosilk solution (n = 8). Nanosilk showed improved biomechanical properties with increased **(A)** maximum load and **(B)** modulus, with no change in **(C)** extension at point of breaking. *p < 0.05.

We compared the biomechanical properties of maximum stress and modulus in human skin samples treated with PBS as control and 7% nanosilk solution. Our data demonstrated that skin samples treated with 7% nanosilk showed improved strength with increased maximum load of 51.47 ± 2.028 N compared to 41.88 ± 3.142 N in samples treated with PBS (p < 0.05, **Figure 7A**). Elastic modulus measures resistance to being deformed when stress is applied. We saw improved modulus with 7% nanosilk “99.67 ± 3.316 MPa” compared to “74.06 ± 9.146 MPa” (p < 0.05, **Figure 7B**) Skin elasticity did not change following the nanosilk treatment (**Figure 7C**).

## Discussion

Diabetic wounds represent a significant health burden for both patients and the healthcare system. Characterized by chronic inflammation, poor wound contraction, and decreased collagen deposition, non-healing diabetic wounds are a leading cause of amputations worldwide. Nanosilk may offer a potential delivery system for novel therapeutics, including CNP-miR146a, while providing improvement in biomechanical strength to diabetic skin already prone to injury.

We have shown in this experiment that nanosilk solution effectively delivers CNP-miR146a to the wound, leading to complete wound closure 2.3 days sooner than sham treated diabetic wounds. This is consistent with our previous data showing intradermal injections of CNP-miR146a accelerates diabetic wound closure (17), and nanosilk solution provides a more clinically accessible treatment option as a topical application rather than an injection. While treatment with nanosilk solution alone did not accelerate wound closure, we did not see any deleterious effects. In fact, there was a reduction in IL-6 relative gene expression seven days into the wound healing process, which is consistent with studies showing that silk fibroin may have some mild anti-inflammatory properties (34). Nanosilk therefore may provide a synergistic effect with CNP-miR146a in wound closure, both in protecting the wound edge due to its intrinsic barrier strength and its anti-inflammatory properties.

When CNP-miR146a is delivered to diabetic wounds in a nanosilk solution, there is a reduction in pro-inflammatory gene expression, specifically IL-6 and IL-8. These inflammatory cytokines are overexpressed in diabetic wounds, preventing recruited macrophages from transitioning from a pro-inflammatory to a pro-resolving phenotype (35). Seven days after injury, this reduction in inflammatory signaling correlates with a reduction in inflammatory cell infiltrate by CD45+ count evaluation, and this cellular infiltrate normalized by the time of full healing. Nanosilk solution alone decreased IL-6 gene expression and had trends toward lower inflammatory cellular infiltrate early in the healing process. Our study may have been underpowered to detect the more modest effects of nanosilk solution alone. We found that CNP-miR146a with nanosilk solution may play an early role in both chemotaxis and in modulating the functionality of the recruited cells in the wound bed. Promoting a pro-resolving macrophage phenotype, for example, could accelerate the transition toward wound closure and remodeling, largely driven by TGFβ-1 signaling and collagen production.

We found that the reduction in IL-6 and IL-8 gene expression 7 days after injury correlated to an increase in TGFβ-1 expression. IL-6, which was decreased by both NS and NS + CNP-miR146a, has been shown to inhibit TGFβ-1 signaling, however the mechanistic pathways in which IL-6 and TGFβ-1 interact in diabetic wounds is not fully understood. IL-6 has been shown to inhibit the differentiation of T-regulatory immune cells through modulation of TGFβ, which play critical roles in wound re-epithelialization (36–38). Furthermore, TGFβ-1 activates fibroblast function, which helps explain the late increase in Col3α1 and Col1α2 gene expression after treatment with NS + CNP-miR146a. TGFβ1 additionally stimulates fibroblast differentiation into myofibroblasts, which participate in wound contracture, and may explain the overall acceleration in wound closure due to CNP-miR146a when given in a nanosilk solution. We have previously shown that diabetic skin is biomechanically inferior to non-diabetic skin with associated reductions in collagen protein levels (39). Consistent with increased collagen gene expression, fully healed diabetic wounds had significantly higher levels of collagen on histologic examination after treating with NS + CNP-miR146a as compared to controls. Not only did the wounds close faster, but the wounds may have improved strength given the increased collagen levels.

Finally, when evaluating the potential of nanosilk solution in human application, we see that there is a biomechanical benefit in the application of nanosilk to uninjured diabetic skin, which is prone to injury. Silk fibroin has been shown to promote cell adhesion and improve type I collagen spread within epidermal keratinocytes and fibroblasts (40). We have previously shown that injection with CNP-miR146a alone did not improve the biomechanical properties of diabetic skin (17). In this study, however, we saw a significant improvement in maximum load and modulus compared to untreated skin after a one-time application of 7% nanosilk solution. This skin strengthening could have potential implications in protecting fragile diabetic skin that is prone to damage. When combined with a therapeutic like CNP-miR146a, nanosilk solution would have the capacity to protect the wound edge from additional injury while the conjugate treatment reduces inflammation and accelerates wound closure.

This experiment shows that nanosilk solution is able to improve the biomechanical properties of diabetic skin, and when given as a topical therapeutic alone to diabetic wounds causes no worsening of the injury while having some anti-inflammatory effect. Nanosilk solution effectively delivers CNP-miR146a to the wound bed, which in turn lowers pro-inflammatory signaling and increases pro-fibrotic processes, ultimately accelerating wound closure.

## Data Availability Statement

The datasets presented in this study can be found in online repositories. The names of the repository/repositories and accession number(s) can be found below: https://figshare.com/, 10.6084/m9.figshare.12749630.

## Ethics Statement

The animal study was reviewed and approved by University of Colorado Denver Institutional Animal Care and Use Committee (IACUC).

## Author Contributions

SN, AL, SH, JX, SSi, KL, and CZ participated in study conception and design. SN, AL, SH, LD, LZ, MA, SSi, and TS performed the acquisition and analysis of data. SN drafted the main manuscript text. All authors contributed to the article and approved the submitted version.

## Funding

This work was supported by the National Institute of Health T32 training grant #AR7411-35 and through Institutional Support.

## Conflict of Interest

CZ and KL hold financial interest and stock in Ceria Therapeutics.

The remaining authors declare that the research was conducted in the absence of any commercial or financial relationships that could be construed as a potential conflict of interest.

## References

[1] Centers for Disease Control and Prevention National Diabetes Statistics Report, 2020. Atlanta, GA: Centers for Disease Control and Prevention, U.S. Dept of Health and Human Services (2020).

[2] Juster-Switlyk K Smith AG Updates in diabetic peripheral neuropathy. F1000Res (2016) 5:1–7. 10.12688/f1000research.7898.1 PMC4847561 27158461

[3] Saran R Robinson B Abbott KC Agodoa LY Albertus P Ayanian J US Renal Data System 2016 Annual Data Report: Epidemiology of Kidney Disease in the United States. Am J Kidney Dis (2017) 69(3 Suppl 1):A7–8. 10.1053/j.ajkd.2017.01.036 PMC6605045 28236831

[4] Shah AD Langenberg C Rapsomaniki E Denaxas S Pujades-Rodriguez M Gale CP Type 2 diabetes and incidence of cardiovascular diseases: a cohort study in 1.9 million people. Lancet Diabetes Endocrinol (2015) 3(2):105–13. 10.1016/S2213-8587(14)70219-0 PMC4303913 25466521

[5] Brem H Tomic-Canic M Cellular and molecular basis of wound healing in diabetes. J Clin Invest (2007) 117(5):1219–22. 10.1172/JCI32169 PMC1857239 17476353

[6] Richard JL Schuldiner S [Epidemiology of diabetic foot problems]. Rev Med Interne (2008) 29 Suppl 2:S222–30. 10.1016/S0248-8663(08)73949-3 18822247

[7] Rice JB Desai U Cummings AK Birnbaum HG Skornicki M Parsons NB Burden of diabetic foot ulcers for medicare and private insurers. Diabetes Care (2014) 37(3):651–8. 10.2337/dc13-2176 24186882

[8] Mirza RE Koh TJ Contributions of cell subsets to cytokine production during normal and impaired wound healing. Cytokine (2015) 71(2):409–12. 10.1016/j.cyto.2014.09.005 PMC4297569 25281359

[9] Maxson S Lopez EA Yoo D Danilkovitch-Miagkova A Leroux MA Concise review: role of mesenchymal stem cells in wound repair. Stem Cells Transl Med (2012) 1(2):142–9. 10.5966/sctm.2011-0018 PMC3659685 23197761

[10] Armour A Scott PG Tredget EE Cellular and molecular pathology of HTS: basis for treatment. Wound Repair Regen (2007) 15 Suppl 1:S6–17. 10.1111/j.1524-475X.2007.00219.x 17727469

[11] Wang J Dodd C Shankowsky HA Scott PG Tredget EE Wound Healing Research G Deep dermal fibroblasts contribute to hypertrophic scarring. Lab Invest (2008) 88(12):1278–90. 10.1038/labinvest.2008.101 18955978

[12] Hehenberger K Kratz G Hansson A Brismar K Fibroblasts derived from human chronic diabetic wounds have a decreased proliferation rate, which is recovered by the addition of heparin. J Dermatol Sci (1998) 16(2):144–51. 10.1016/S0923-1811(97)00042-X 9459127

[13] Loots MA Lamme EN Mekkes JR Bos JD Middelkoop E Cultured fibroblasts from chronic diabetic wounds on the lower extremity (non-insulin-dependent diabetes mellitus) show disturbed proliferation. Arch Dermatol Res (1999) 291(2-3):93–9. 10.1007/s004030050389 10195396

[14] Seibold JR Uitto J Dorwart BB Prockop DJ Collagen synthesis and collagenase activity in dermal fibroblasts from patients with diabetes and digital sclerosis. J Lab Clin Med (1985) 105(6):664–7. 2987379

[15] Moura J Borsheim E Carvalho E The Role of MicroRNAs in Diabetic Complications-Special Emphasis on Wound Healing. Genes (Basel) (2014) 5(4):926–56. 10.3390/genes5040926 PMC4276920 25268390

[16] Rafehi H El-Osta A Karagiannis TC Epigenetic mechanisms in the pathogenesis of diabetic foot ulcers. J Diabetes Complications (2012) 26(6):554–61. 10.1016/j.jdiacomp.2012.05.015 22739801

[17] Zgheib C Hilton SA Dewberry LC Hodges MM Ghatak S Xu J Use of Cerium Oxide Nanoparticles Conjugated with MicroRNA-146a to Correct the Diabetic Wound Healing Impairment. J Am Coll Surg (2019) 228(1):107–15. 10.1016/j.jamcollsurg.2018.09.017 PMC7846138 30359833

[18] Das S Dowding JM Klump KE McGinnis JF Self W Seal S Cerium oxide nanoparticles: applications and prospects in nanomedicine. Nanomed (Lond) (2013) 8(9):1483–508. 10.2217/nnm.13.133 23987111

[19] Dowding JM Dosani T Kumar A Seal S Self WT Cerium oxide nanoparticles scavenge nitric oxide radical (NO). Chem Commun (Camb) (2012) 48(40):4896–8. 10.1039/c2cc30485f 22498787

[20] Grulke E Reed K Beck M Huang X Cormack A Seal S Nanoceria: factors affecting its pro- and anti-oxidant properties. Environ Sci-Nano (2014) 1(5):429–44. 10.1039/C4EN00105B

[21] Heckert EG Karakoti AS Seal S Self WT The role of cerium redox state in the SOD mimetic activity of nanoceria. Biomaterials (2008) 29(18):2705–9. 10.1016/j.biomaterials.2008.03.014 PMC2396488 18395249

[22] Karakoti AS Monteiro-Riviere NA Aggarwal R Davis JP Narayan RJ Self WT Nanoceria as Antioxidant: Synthesis and Biomedical Applications. JOM (1989) 60(3):33–7. 2008. 10.1007/s11837-008-0029-8 PMC2898180 20617106

[23] Korsvik C Patil S Seal S Self WT Superoxide dismutase mimetic properties exhibited by vacancy engineered ceria nanoparticles. Chem Commun (Camb) (2007) 10:1056–8. 10.1039/b615134e 17325804

[24] Chigurupati S Mughal MR Okun E Das S Kumar A McCaffery M Effects of cerium oxide nanoparticles on the growth of keratinocytes, fibroblasts and vascular endothelial cells in cutaneous wound healing. Biomaterials (2013) 34(9):2194–201. 10.1016/j.biomaterials.2012.11.061 PMC3552035 23266256

[25] Colon J Hsieh N Ferguson A Kupelian P Seal S Jenkins DW Cerium oxide nanoparticles protect gastrointestinal epithelium from radiation-induced damage by reduction of reactive oxygen species and upregulation of superoxide dismutase 2. Nanomedicine (2010) 6(5):698–705. 10.1016/j.nano.2010.01.010 20172051

[26] Das S Singh S Dowding JM Oommen S Kumar A Sayle TX The induction of angiogenesis by cerium oxide nanoparticles through the modulation of oxygen in intracellular environments. Biomaterials (2012) 33(31):7746–55. 10.1016/j.biomaterials.2012.07.019 PMC4590782 22858004

[27] Dowding JM Song W Bossy K Karakoti A Kumar A Kim A Cerium oxide nanoparticles protect against Abeta-induced mitochondrial fragmentation and neuronal cell death. Cell Death Differ (2014) 21(10):1622–32. 10.1038/cdd.2014.72 PMC4158687 24902900

[28] Bhaumik D Scott GK Schokrpur S Patil CK Orjalo AV Rodier F MicroRNAs miR-146a/b negatively modulate the senescence-associated inflammatory mediators IL-6 and IL-8. Aging (Albany NY) (2009) 1(4):402–11. 10.18632/aging.100042 PMC2818025 20148189

[29] Tang L Li X Bai Y Wang P Zhao Y MicroRNA-146a negatively regulates the inflammatory response to Porphyromonas gingivalis in human periodontal ligament fibroblasts via TRAF6/p38 pathway. J Periodontol (2019) 90(4):391–9. 10.1002/JPER.18-0190 30378773

[30] Xie W Tadepalli S Park SH Kazemi-Moridani A Jiang Q Singamaneni S Extreme Mechanical Behavior of Nacre-Mimetic Graphene-Oxide and Silk Nanocomposites. Nano Lett (2018) 18(2):987–93. 10.1021/acs.nanolett.7b04421 29314859

[31] Bermudez DM Herdrich BJ Xu J Lind R Beason DP Mitchell ME Impaired biomechanical properties of diabetic skin implications in pathogenesis of diabetic wound complications. Am J Pathol (2011) 178(5):2215–23. 10.1016/j.ajpath.2011.01.015 PMC3081147 21514435

[32] Rockwood DN Preda RC Yucel T Wang X Lovett ML Kaplan DL Materials fabrication from Bombyx mori silk fibroin. Nat Protoc (2011) 6(10):1612–31. 10.1038/nprot.2011.379 PMC3808976 21959241

[33] Singh S Ly A Das S Sakthivel TS Barkam S Seal S Cerium oxide nanoparticles at the nano-bio interface: size-dependent cellular uptake. Artif Cells Nanomed Biotechnol (2018) 46(sup3):S956–S63. 10.1080/21691401.2018.1521818 30314412

[34] Rodriguez-Nogales A Lozano-Perez AA Aznar-Cervantes SD Algieri F Garrido-Mesa J Garrido-Mesa N Effect of aqueous and particulate silk fibroin in a rat model of experimental colitis. Int J Pharm (2016) 511(1):1–9. 10.1016/j.ijpharm.2016.06.120 27363935

[35] Hesketh M Sahin KB West ZE Murray RZ Macrophage Phenotypes Regulate Scar Formation and Chronic Wound Healing. Int J Mol Sci (2017) 18(7):1534–45. 10.3390/ijms18071545 PMC5536033 28714933

[36] Johnson BZ Stevenson AW Prele CM Fear MW Wood FM The Role of IL-6 in Skin Fibrosis and Cutaneous Wound Healing. Biomedicines (2020) 8(5):1–18. 10.3390/biomedicines8050101 PMC7277690 32365896

[37] Kuhn C Rezende RM M’Hamdi H da Cunha AP Weiner HL IL-6 Inhibits Upregulation of Membrane-Bound TGF-beta 1 on CD4+ T Cells and Blocking IL-6 Enhances Oral Tolerance. J Immunol (2017) 198(3):1202–9. 10.4049/jimmunol.1600921 PMC5463579 28039301

[38] Haertel E Joshi N Hiebert P Kopf M Werner S Regulatory T cells are required for normal and activin-promoted wound repair in mice. Eur J Immunol (2018) 48(6):1001–13. 10.1002/eji.201747395 29457218

[39] Zgheib C Hodges M Hu J Beason DP Soslowsky LJ Liechty KW Mechanisms of mesenchymal stem cell correction of the impaired biomechanical properties of diabetic skin: The role of miR-29a. Wound Repair Regener (2016) 24(2):237–46. 10.1111/wrr.12412 PMC7691865 26808714

[40] Min BM Lee G Kim SH Nam YS Lee TS Park WH Electrospinning of silk fibroin nanofibers and its effect on the adhesion and spreading of normal human keratinocytes and fibroblasts in vitro. Biomaterials (2004) 25(7-8):1289–97. 10.1016/j.biomaterials.2003.08.045 14643603

